# The immunostimulatory effect of indole-6-carboxaldehyde isolated from *Sargassum thunbergii* **(Mertens) Kuntze** in RAW 264.7 macrophages

**DOI:** 10.1080/19768354.2020.1808529

**Published:** 2020-08-20

**Authors:** Cheol Park, Hyun HwangBo, Hyesook Lee, Gi-Young Kim, Hee-Jae Cha, Sung Hyun Choi, Suhkmann Kim, Heui-Soo Kim, Seok Joong Yun, Wun-Jae Kim, You-Jin Jeon, Yung Hyun Choi

**Affiliations:** a Division of Basic Sciences, College of Liberal Studies, Dong-eui University, Busan, Republic of Korea; b Anti-Aging Research Center, Dong-eui University, Busan, Republic of Korea; c Department of Biochemistry, Dong-eui University College of Korean Medicine, Busan, Republic of Korea; d Department of Marine Life Sciences, School of Marine Biomedical Sciences, Jeju National University, Jeju, Republic of Korea; e Department of Parasitology and Genetics, Kosin University College of Medicine, Busan, Republic of Korea; f Department of System Management, Korea Lift College, Geochang, Republic of Korea; g Department of Chemistry, College of Natural Sciences, Pusan National University, Busan, Republic of Korea; h Department of Biological Sciences, College of Natural Sciences, Pusan National University, Busan, Republic of Korea; i Department of Urology, College of Medicine, Chungbuk National University, Cheongju, Republic of Korea

**Keywords:** Indole-6-carboxaldehyde, immunomodulatory property, TLR4, NF-κB

## Abstract

Indole-6-carboxaldehyde (I6CA), an indole derivative isolated from the marine brown algae *Sargassum thunbergii*, is known to have several beneficial effects, but no studies on immune regulation have been conducted. In this study, the immunomodulatory properties of I6CA on murine RAW 264.7 monocyte/macrophage cells were evaluated. As the concentration of I6CA increased, the morphology of RAW 264.7 cells changed to a typical active macrophage shape, and the phagocytic activity increased significantly. I6CA effectively enhanced the production and secretion of immunomodulatory mediators and cytokines due to increased expression of their respective genes. Additionally, I6CA markedly stimulated the expression of Toll-like receptor 4 (TLR4) and its adapter molecule, myeloid differentiation factor 88 (Myd88), and increased TLR4 complexed with Myd88. Furthermore, I6CA promoted the nuclear translocation of nuclear factor-kappa B (NF-κB) by increasing the degradation of the inhibitor of NF-κB-*α*. Meanwhile, similar trends were also found in lipopolysaccharide-treated cells as a positive control. Furthermore, molecular docking simulation showed that I6CA interacted with TLR4-myeloid differentiation 2 complex. Taken together, the results support the concept that I6CA may increase the activity of the TLR4/NF-κB signaling pathway in order to enhance the immunomodulatory activity of RAW 264.7 cells.

## Introduction

Macrophages are the main immune cells that act as effector cells to fight against inflammation or initiating innate immune responses. They originate from blood monocytes that leave the circulatory system and differentiate in other tissues (Ma et al. 2019; Domínguez-Andrés et al. 2020). Monocytes are activated by multiple biological substances as well as microbial components, including lipopolysaccharide (LPS), a kind of membrane component of Gram-negative bacteria, which is recognized by Toll-like receptors (TLRs) and triggers several signaling pathways to activate macrophages (Shapouri-Moghaddam et al. 2018; Domínguez-Andrés et al. 2020). Consequently, foreign substances and cellular debris can be directly eliminated through phagocytosis, the cornerstone of the innate immune response. Activated macrophages also neutralize pathogens through the secretion of inflammatory mediators and cytokines or indirectly participate in immune regulation. In addition, they increase immune-enhancing activity through the processing and presenting antigens to lymphocytes. Although various drugs are currently used to regulate immune function to prevent and treat chronic diseases, their use is limited because they are mostly toxic or have side effects (Gupta and Prakash 2014; Martinez and Peplow 2020). Therefore, the activation of macrophages using non-toxic natural products is considered a promising strategy to improve host immune function.

Indole and its derivatives having an aromatic heterocyclic structure are active molecules formed from naturally occurring glucosinolates (Prieto et al. 2019). Many previous studies have shown that indole derivatives have attracted much attention due to their potential health-enhancing properties, including immunomodulatory (Arreola et al. 2015; Bai et al. 2017). Recently, in the process of exploring novel indole derivatives with anti-obesity effects, Kang et al. (2017) found that indole-6-carboxaldehyde (I6CA) isolated from the marine brown algae *Sargassum thunbergii* (Mertens) Kuntze is an effective candidate. In addition, Kim et al. (2019a) reported that I6CA suppressed tumor invasion and metastasis by inhibiting the activity of matrix metalloproteinase-9 and preventing degradation of the extracellular matrix. However, there is a lack of evidence to describe the underlying mechanisms and limited information available about whether I6CA can enhance immune function. Therefore, in this study, we evaluated the immunomodulatory effect of I6CA using a murine RAW 264.7 monocyte/macrophage cell model.

## Materials and methods

### Cell culture

RAW 264.7 cells were grown in Dulbecco’s modified Eagle’s medium (DMEM) supplemented with 10% fetal bovine serum and antibiotics mixture (WelGENE Inc., Gyeongsan, Republic of Korea) at 37°C in a humidified atmosphere of 5% CO_2_. I6CA (Sigma-Aldrich Chemical Co., St. Louis, MO, USA) was dissolved in dimethyl sulfoxide (DMSO), and diluted with DMEM to adjust the final treatment concentrations before use in experiments.

### Cell viability assay

RAW 264.7 cells were treated with a series of I6CA concentrations or LPS (Sigma-Aldrich Chemical Co). After 24 h of incubation, 3-(4,5-dimethylthiazol-2-yl)-2,5-diphenyltetra-zolium bromide (MTT) assay was performed as previously described (Hasnn et al. 2019). The LPS-treated cells were considered a positive control for activating the macrophages. The morphologic changes were observed under a phase-contrast microscope (Carl Zeiss, Oberkochen, Germany).

### Phagocytic activity assay

To evaluate the phagocytic activity, a phagocytosis assay kit purchased from Cayman Chemical (Ann Arbor, MI, USA) was used. Following manufacturer’s instructions, the cells were treated with the indicated concentrations of I6CA or LPS for 24 h. Subsequently, latex bead-rabbit IgG-fluorescein isothiocyanate (FITC) complexes were added to the medium (final dilution, 1:200) for 2 h. The cells were washed using the assay buffer provided in the kit. The fluorescence intensity was measured using a flow cytometer (BD Biosciences, San Jose, CA, USA).

### Determination of NO, PGE_2_ and cytokine production assay

After treatment with I6CA or LPS for 24 h, the collected culture supernatants were mixed with an equal volume of Griess reagent solution (Sigma-Aldrich Chemical Co.), and incubated for 30 min at room temperature (RT). The NO concentrations were measured with a microplate reader at 540 nm by referencing a standard curve generated with known concentrations of sodium nitrite. The levels of prostaglandin E_2_ (PGE_2_) and cytokines, including tumor necrosis factor (TNF)-*α*, interleukin (IL)-1*β*, IL-6, and IL-10, were quantified using the corresponding commercial enzyme-linked immunosorbent assay (ELISA) kits (R&D Systems, Inc., Minneapolis, MN, USA) according to the vender’s instructions. Standard curves prepared from standard samples were used to calculate the PGE_2_ and cytokine production.

### Reverse transcriptase-polymerase chain reaction (RT–PCR)

Total RNA extracted using a total RNA extraction kit was reverse-transcribed using M-MLV reverse transcriptase (Bioneer, Daejeon, Republic of Korea). Subsequently, the PCR products obtained by amplifying the target cDNA using the primers of the desired genes were electrophoresed using 1.5% agarose gel (Kim et al. 2019b). The gels were stained with ethidium bromide and visualized by ultraviolet illumination.

### Western blot analysis

The cells were lysed with lysis buffer containing cellular protease inhibitor and phosphatase inhibitor as previously described (Hong et al. 2019). The nuclear and cytosolic proteins were extracted using a nuclear extract kit (Active Motif, Inc., Carlsbad, CA, USA). For co-immunoprecipitation (Co-IP) assay, the lysate was diluted with lysis buffer. After immunoprecipitation using anti-TLR4 antibody (Santa Cruz Biotechnology, Inc., Santa Cruz, CA, USA) for 12 h at 4°C, the immune-complexes were recovered with protein A-agarose beads (Sigma-Aldrich Chemical Co.) for 2 h at 4°C. After denaturation, equal amounts of protein or immune-complexes were separated by sodium-dodecyl sulfate-polyacrylamide gel electrophoresis and then transferred onto polyvinylidene difluoride membranes (Merck Millipore, Bedford, MA, USA). The membranes were probed with corresponding primary antibodies overnight at 4°C while shaking. Subsequently, the strips were incubated with the appropriate horseradish peroxidase-conjugated antibodies (Santa Cruz Biotechnology, Inc.) at RT for 2 h, and then visualized using an enhanced chemiluminescence detection system (R&D Systems, Inc.).

### Molecular docking of I6CA with TLR4/myeloid differentiation 2 (MD2) complex

The docking of I6CA with TLR4/MD2 was simulated using the PyRx virtual screening program (https://pyrx.sourceforge.io), which has been used to evaluate the conformation of protein–ligand complexes. The structure of TLR4/MD2 was retrieved from the protein data bank (PDB), and the I6CA structure was obtained from the National Center for Biotechnology Information PubChem that is as chemical information repository. The PDB ID and PubChem compound identifier (CID) were shown in Table 1. The binding affinity of the complex was analyzed by PyMOL molecular graphics system (https://pymol.org).

**Table 1.** Binding information of I6CA and TLR4/MD2 complex.

Molecule	PDB ID	Ligand	PubChem ID	Binding affinity (kcal/mol)	Binding site
TLR4	3VQ2	Indole-6-carboxaldehyde	CID 24882323	−6.2	O (Lys 132)

### Immunofluorescent staining for NF-κB

The cells were seeded on coverslips and allowed to attach overnight and then treated with I6CA or LPS for 1 h. The cells were fixed with 3.7% paraformaldehyde for 10 min at 4°C and permeabilized with 0.4% Triton X-100 at RT for 10 min. The cells were blocked with 5% bovine serum albumin for 1 h and probed with anti-NF-κB/p65 antibody overnight at 4°C. After washing with PBS, FITC-conjugated donkey anti-rabbit IgG (Sigma-Aldrich Chemical Co.) was added for 2 h at RT. Then, the cells were stained with 0.5 mg/ml 4,6-diamidino-2-phenylindole (DAPI) solution for 10 min to determine the position of nucleus. The fluorescence images were captured using a fluorescence microscope (Carl Zeiss).

### Statistical analysis

The results were expressed as the mean ± deviation (SD). Statistical analyses were performed using the SPSS software, version 16.0 (SPSS Inc., Chicago, IL, USA). The statistical significance was analyzed by one-way ANOVA. *p *< 0.05 was taken as statistically significant.

## Results

### Effect of I6CA on RAW 264.7 cell viability

**Figure 1.**
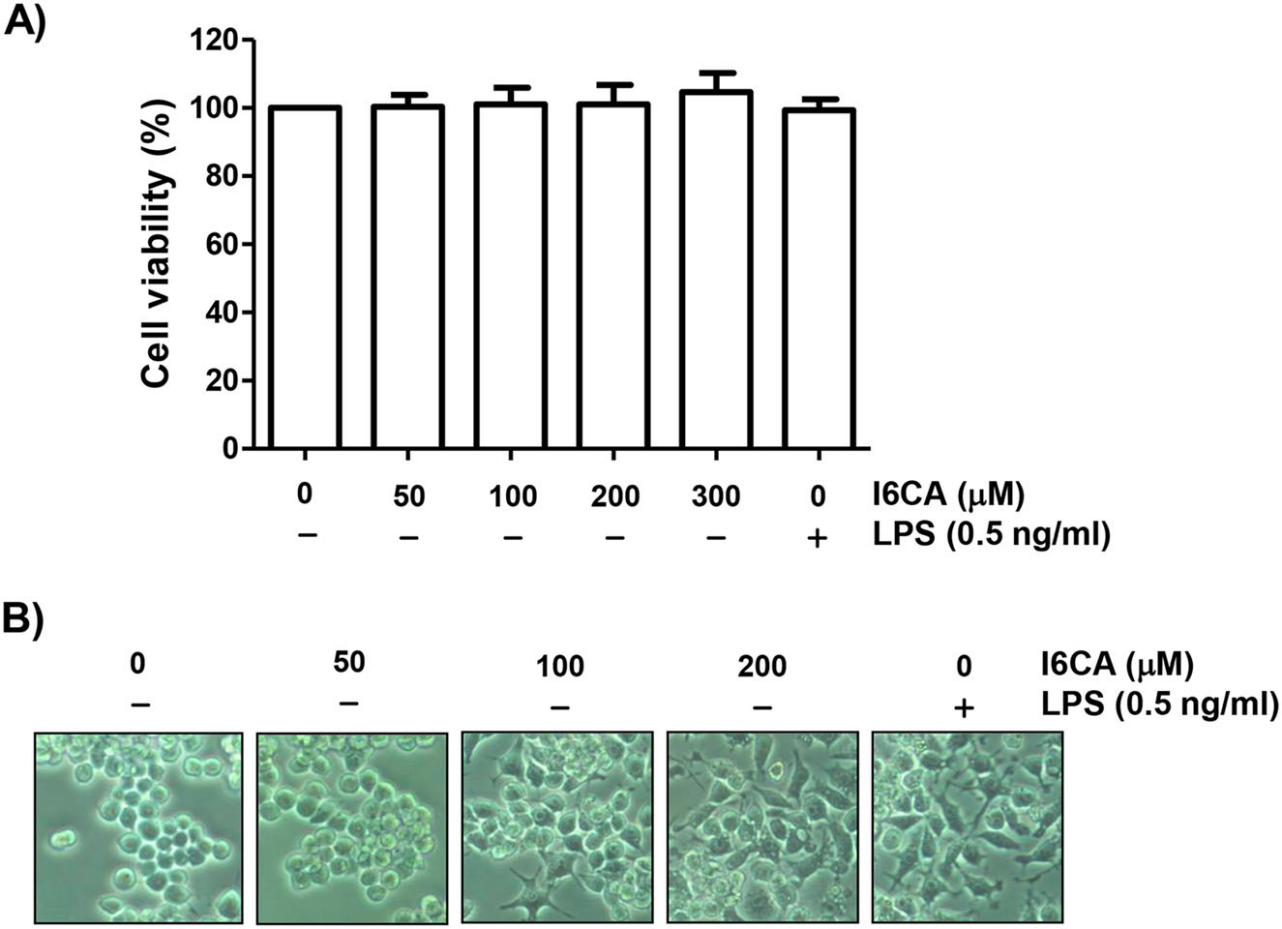
Effect of I6CA on the viability of RAW 264.7 cells. The cells were treated with the indicated concentrations of I6CA or LPS for 24 h. (A) Cell viability was assessed by an MTT reduction assay and the results are expressed as the mean ± SD of three independent experiments. (B) Representative pictures of the morphological changes are shown (200 × original magnification).

As indicated in Figure 1(A), RAW 264.7 cells treated with I6CA up to 300 μM show no significant difference in cell viability compared to control. Therefore, I6CA concentrations of up to 200 μM were selected for the subsequent experiments. LPS at a concentration of 0.5 ng/ml, used as a positive control to activate the macrophages, also showed no cytotoxicity to RAW 264.7 cells.

### I6CA increased the phagocytic activity of RAW 264.7 cells

**Figure 2.**
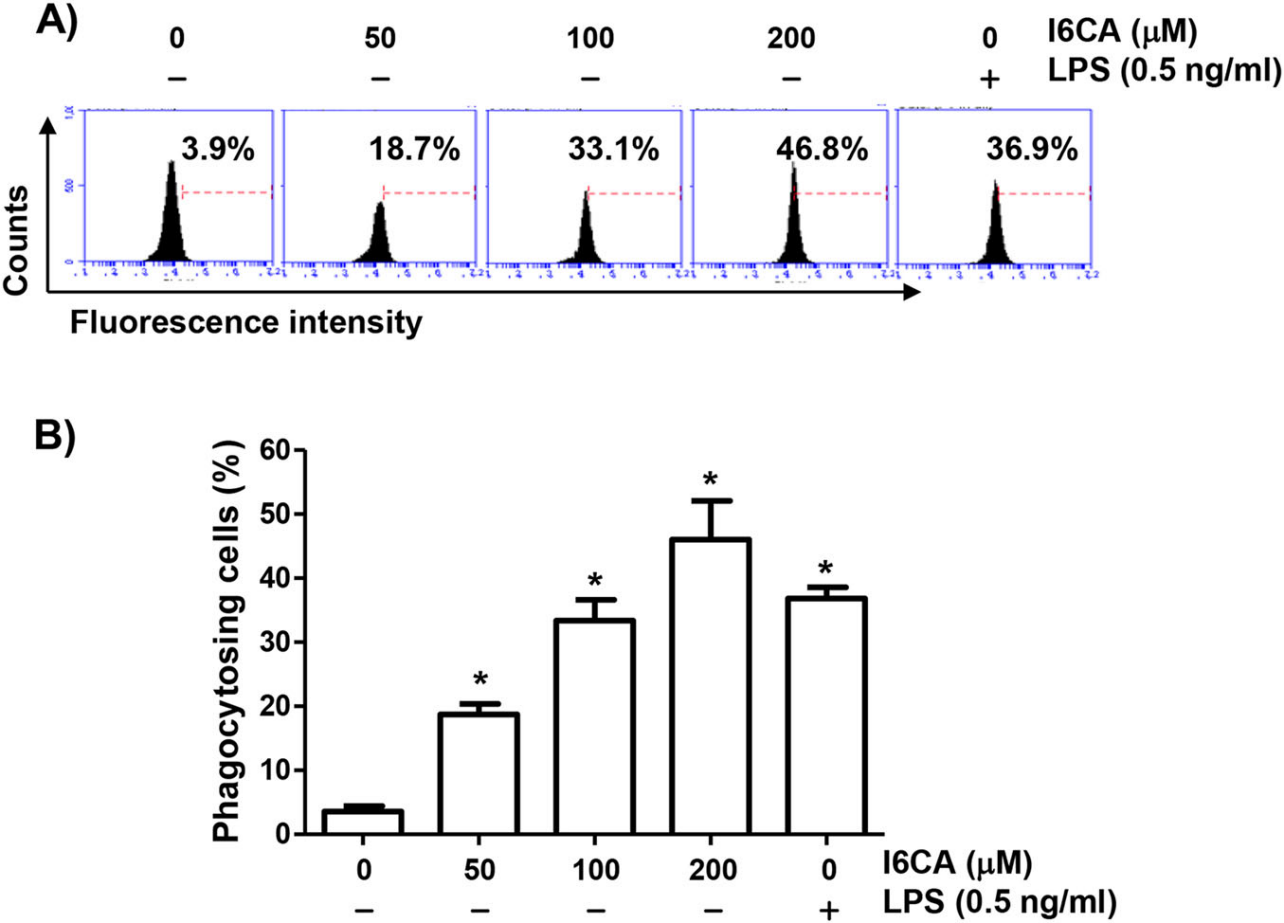
Increased phagocytic activity of RAW 264.7 cells by I6CA. The cells were treated with I6CA or LPS for 24 h. (A) Phagocytic activity of I6CA was measured using flow cytometry. Representative profiles. (B) The percentages of cells that phagocytosed beads are indicated. All experiments were repeated three times (^#^*p *< 0.05 compared to the control group).

Because morphological changes of macrophages can be influenced by various exogenous factors and sometimes related to their activation, we investigated the effect of I6CA on the morphology of RAW 264.7 cells. RAW 264.7 cells cultured in normal medium were generally round or oval. However, with increasing I6CA treatment concentration, the cells became irregular, large, diamond-shaped, and the surface became rough and crude (Figure 1(B)). As a positive control, exposure of cells to LPS tended to cause similar morphological changes. To investigate whether I6CA affected the phagocytic activity, we applied flow cytometry analysis and confirmed that I6CA significantly enhanced phagocytosis (Figure 2). The phagocytic activity of cells treated with 100 μM I6CA was similar to that of LPS-treated cells, indicating that I6CA was able to activate RAW 264.7 cells.

### I6CA induced the production of NO and PGE_2_

**Figure 3.**
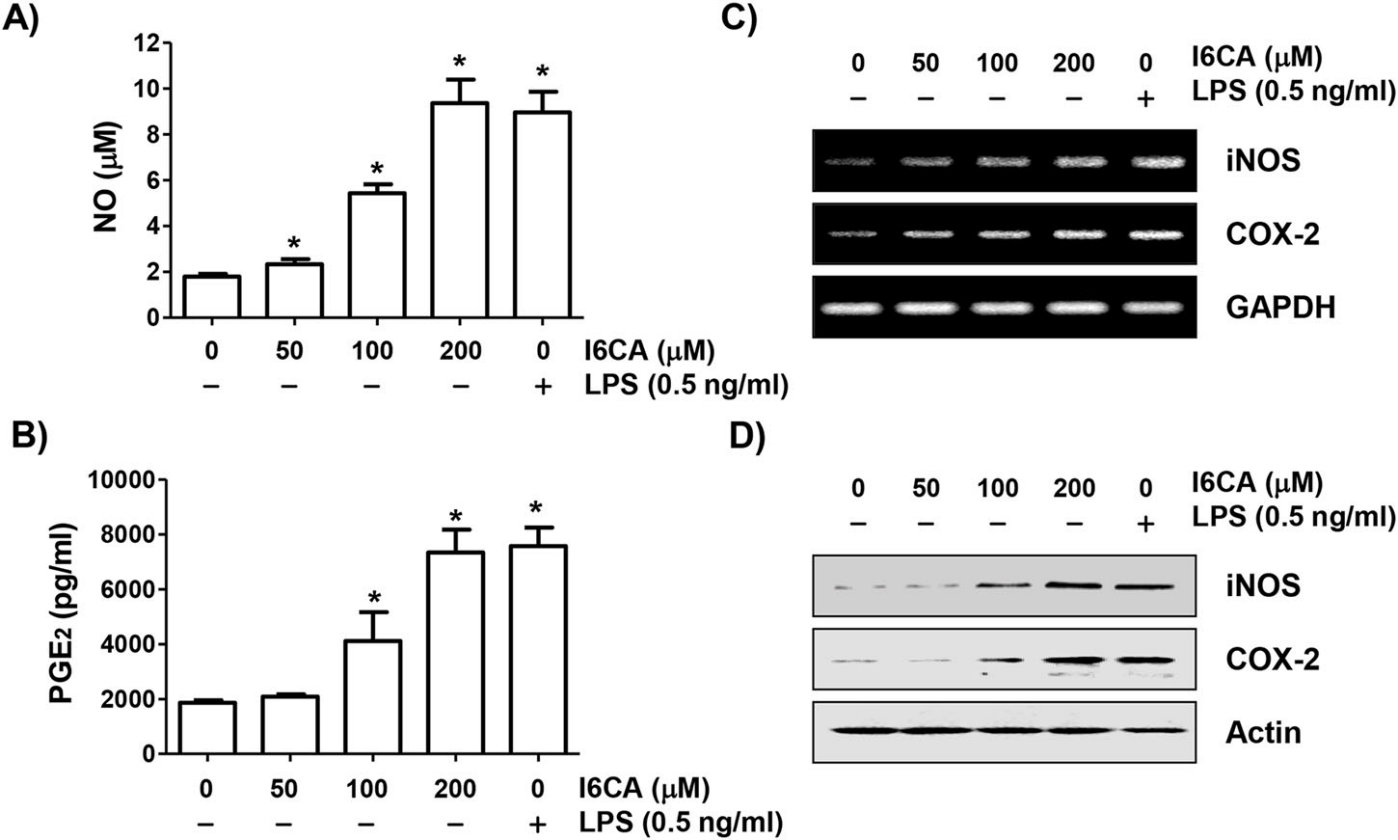
Induction of NO and PGE_2_ production in I6CA-treated RAW 264.7 cells. The cells were treated with I6CA or LPS for 24 h. The levels of NO (A) and PGE_2_ (B) in culture media were measured. Data are presented as the means ± SD obtained from three independent experiments (^#^*p *< 0.05 compared to the control group). The expressions of iNOS and COX-2 mRNA (C) and protein (D) were detected by RT-PCR and immunoblotting. Glyceraldehyde 3-phosphate dehydrogenase (GAPDH) and actin were used as the internal controls for the RT-PCR and immunoblotting, respectively.

To evaluate the effects of I6CA on production of NO and PGE_2_, the concentrations of nitrite and PGE_2_ accumulated in conditioned media were quantified. As indicated in Figure 3(A) and (B), the NO and PGE2 production significantly increased compared to the control group as the I6CA treatment concentration increased. The concentrations produced by the cells treated with 200 μM I6CA were similar to the LPS positive control.

### I6CA enhanced the secretion of cytokines

**Figure 4.**
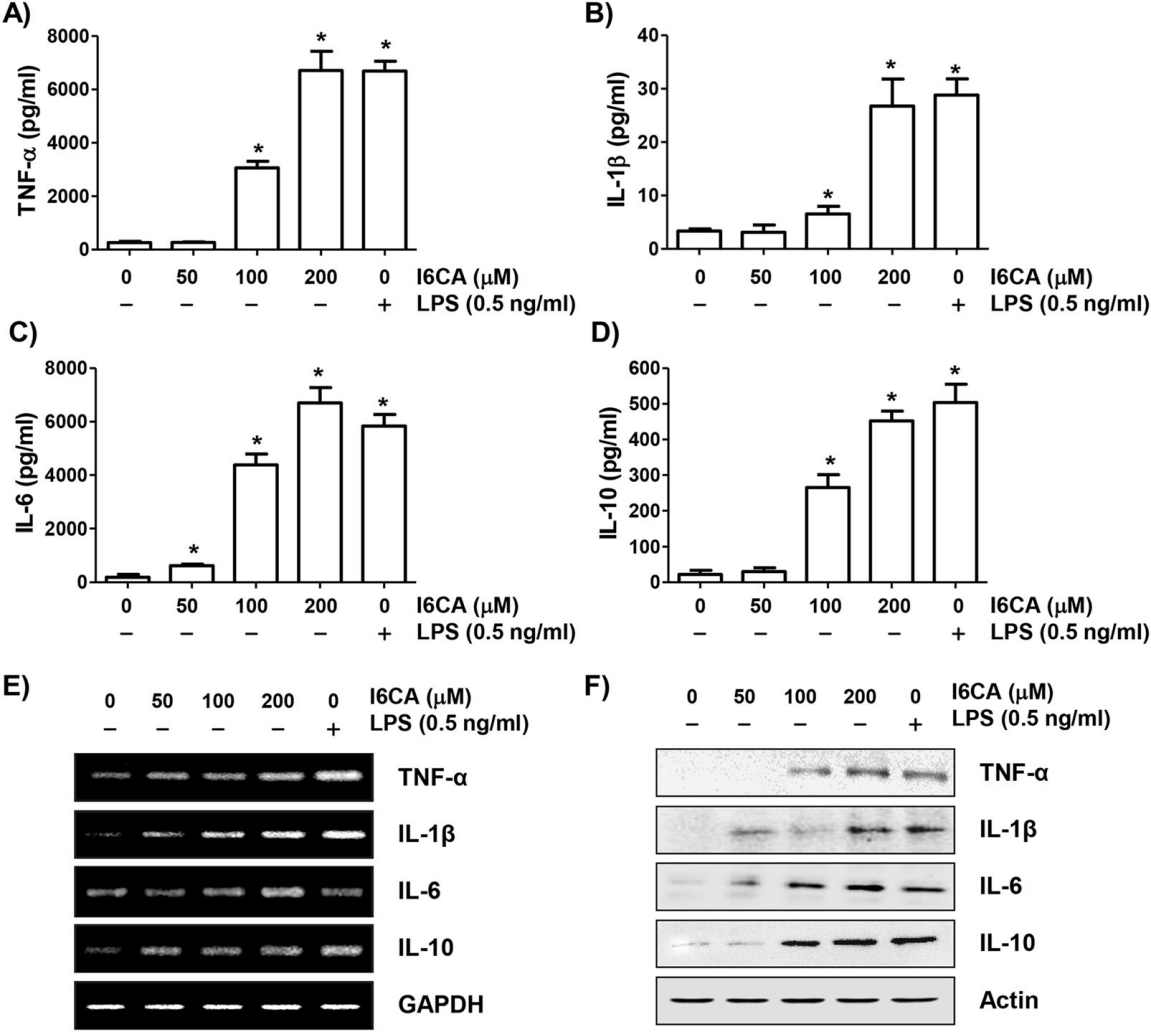
Increased secretion and expression of cytokines by I6CA-treated RAW 264.7 cells. The cells were treated with I6CA or LPS for 24 h. (A-D) Cytokine concentrations were measured by ELISA kits. Data are presented as the means ± SD obtained from three independent experiments (^#^*p *< 0.05 compared to the control group). The expressions of cytokines mRNA (E) and protein (F) were detected by RT-PCR and immunoblotting. GAPDH and actin were used as the internal controls for the RT-PCR and immunoblotting, respectively.

To investigate the effect of I6CA on the secretion of cytokines from RAW 264.7 cells, ELISA assays were performed. As shown in Figures 4(A–D), I6CA significantly enhanced the secretion of all tested cytokines such as TNF-*α*, IL-1*β*, IL-6, and IL-10 compared to that of the control cells in a concentration-dependent manner.

### I6CA promoted the expression of iNOS, COX-2 and cytokines

RT–PCR and immunoblotting results showed that the expression levels of iNOS and COX-2 mRNA and protein were concentration-dependently increased in I6CA-treated cells compared to the control group (Figure 3(C) and (D)). Similar to these results, the expression of cytokines was also increased by treatment with I6CA (Figure 4(E) and (F)). The positive LPS control also significantly increased the expression of all these mRNA and proteins.

### I6CA promoted the expression of TLR4 and Myd88, and interacted the TLR4-MyD88 complex

**Figure 5.**
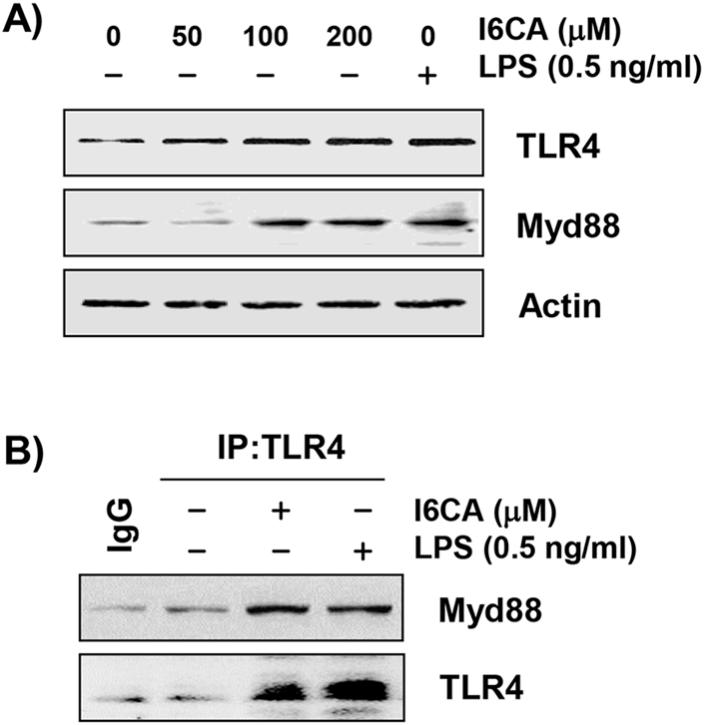
Induction of TLR4 and Myd88 by I6CA in RAW 264.7 cells. The cells were treated with I6CA or LPS for 24 h. (A) The levels of TLR4 and Myd88 protein were evaluated by Western blot analysis. Actin was used as an internal control. (B) Co-IP assay indicated that I6CA-induced TLR4 interacted with Myd88. IgG serves as a negative control of IP.

**Figure 6.**
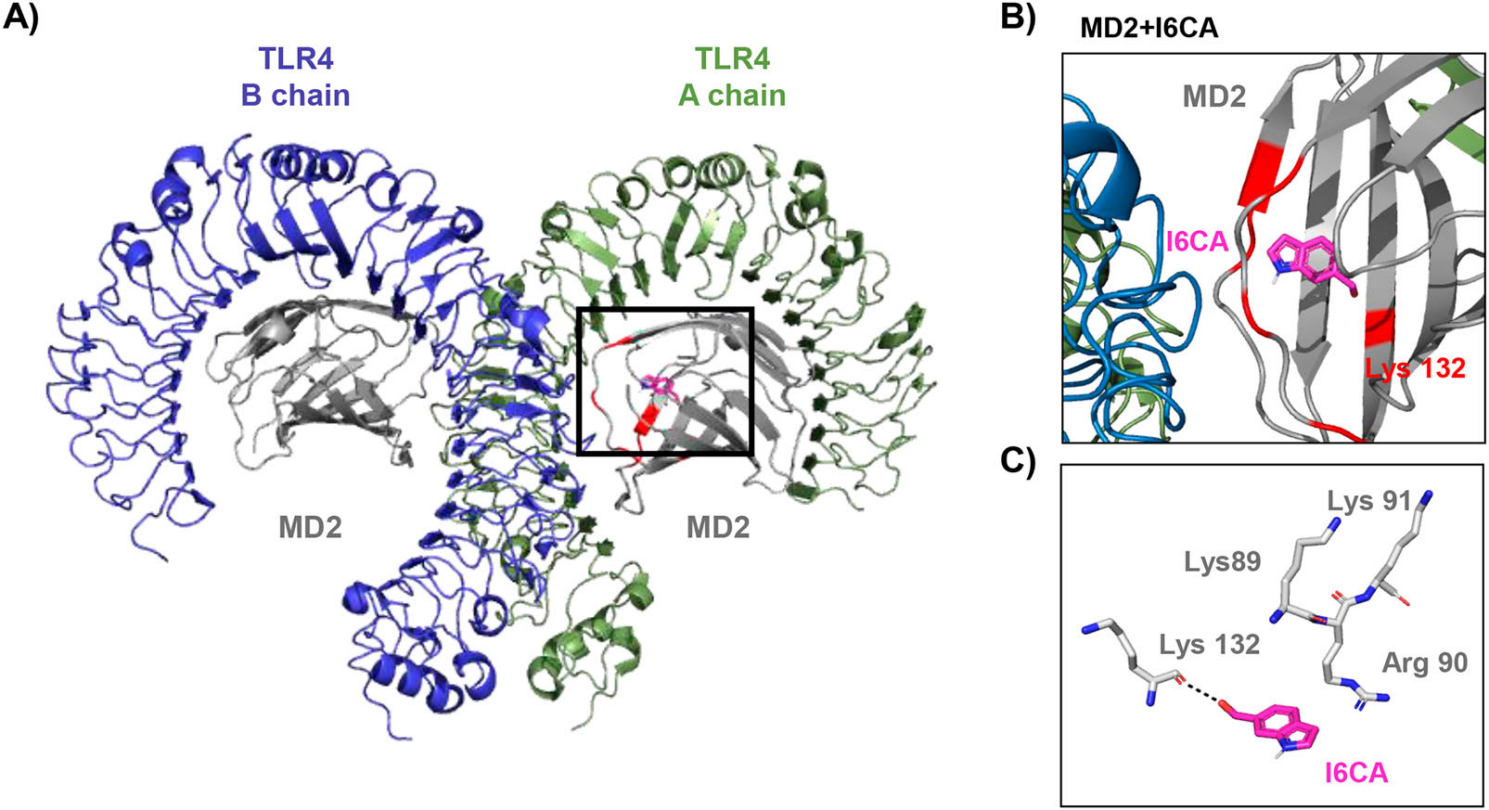
The three-dimensional structure of I6CA and TLR4/MD2 complex. (A and B) Representative configurations of interaction with I6CA and TLR4/MD2 complex. TLR4 chain A, TLR4 chain B, MD2 and I6CA are indicated green, blue, gray and pink stick, respectively. The red colored regions represent the LPS binding residue. (C) The binding distance between I6CA and Lys132 residue of TLR4/MD2 complex.

To examine whether the TLR4/MyD88 signaling pathway was involved in I6CA-induced RAW 264.7 macrophage activation, the levels of TLR4 and MyD88 were measured. Immunoblotting results demonstrated that the protein levels of both proteins in I6CA-treaed RAW 264.7 cells increased concentration-dependently. Their expression in LPS-treated cells was also much higher than in the untreated control. In addition, TLR4 was co-immunoprecipitated with MyD88 after I6CA exposure and, as expected, TLR4 was also immunoprecipitated with MyD88 after LPS treatment (Figure 5). Subsequently, to evaluate whether the interaction with I6CA and TLR4/MD2, a molecular docking study was carried out. The binding affinity of I6CA and TLR4/MD2 complex was −6.2 kcal/mol, and the binding interface was coded by PDB as 3VQ2 (Table 1). Furthermore, I6CA showed the propensity to bind the Lys 132 residue of TLR4-MD2 complex, where is a coordinated covalent bond by LPS (Figure 6).

### I6CA activated the NF-κB signaling pathway

**Figure 7.**
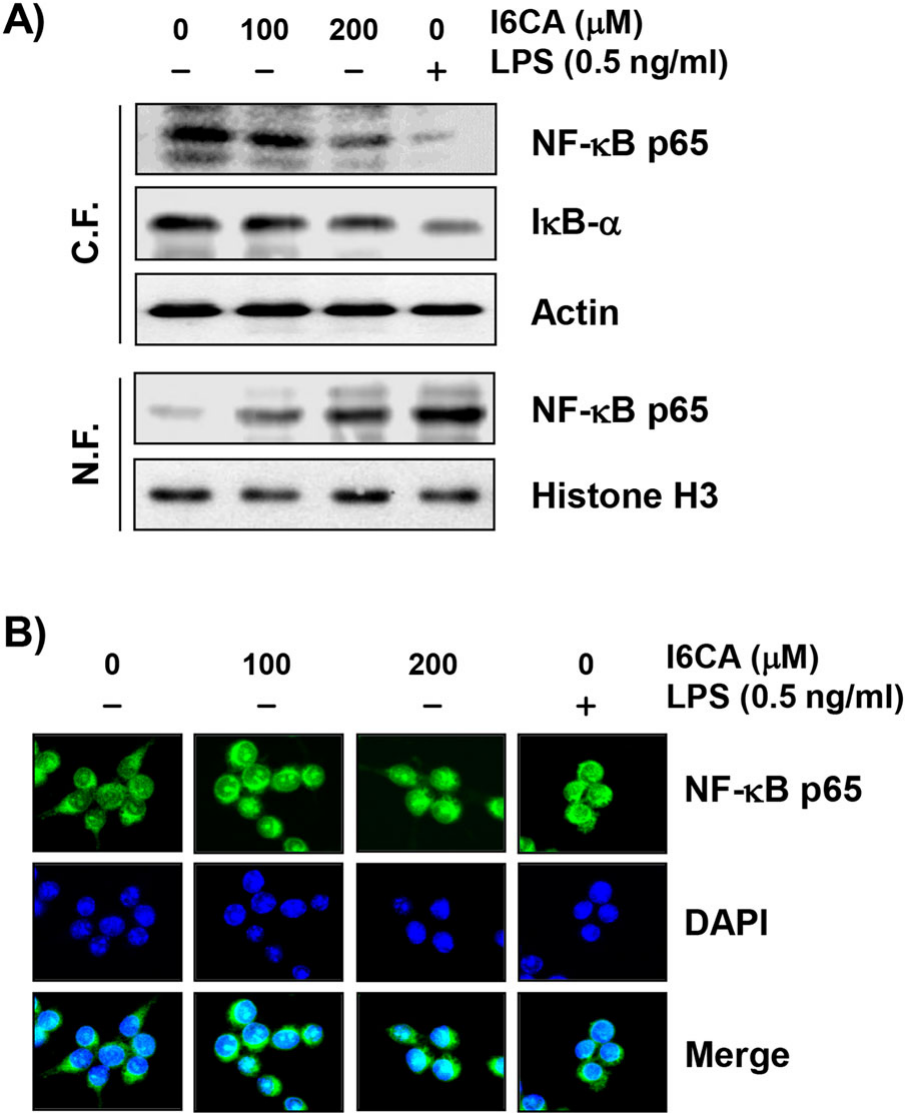
Effect of I6CA on the expression of NF-κB, and IκB-*α*. RAW 264.7 cells were treated with I6CA or LPS for 24 h. (A) Cytoplasmic and nuclear proteins were isolated for analysis of NF-κB, IκB-*α*, and p-IκB-*α* expression, and Western blot analysis was performed. Analysis of actin and histone H3 expression was performed to confirm the protein loading of each fraction extract. (B) The localization of NF-κB/p65 (green) was visualized by fluorescence microscopy. These cells were also stained with DAPI to visualize the nuclei (blue).

To investigate the effect of I6CA on the NF-κB signaling pathway, the expression of NF-κB and IκB-*α* was examined. Figure 7(A) shows that the expression of NF-κB/p65, one important NF-κB subunit for its activation, was up-regulated in the nucleus of I6CA-treated cells and down-regulated in the cytoplasm. The total protein expression of IκB-*α*, a cytoplasmic inhibitor of NF-κB, in the cytosolic fraction was gradually decreased with increasing concentrations of I6CA compared to that in the untreated control group. For further confirmation, the expression position of NF-κB/p65 in the cells was analyzed by fluorescence microscopy. As shown in Figure 7(B), as the concentration of I6CA treatment increased, cytoplasmic NF-κB/p65 was translocated into the nucleus as indicated by the fluorescence intensity of NF-κB/p65. Similar trends were also found in RAW 264.7 cells treated with LPS.

## Discussion

In this study, we evaluated the immunomodulatory activity of I6CA isolated from the marine brown algae *S. thunbergii*, as part of an ongoing screening program for immunostimulants in marine algae. I6CA activated RAW 264.7 cells to secrete NO, PGE_2,_ and cytokines and stimulated the expression of genes responsible for their production. I6CA also increased the expression of TLR4 and MyD88 and activated the NF-κB signaling pathway.

Macrophages can be activated by appropriate stimuli such as bacterial endotoxins and immune-regulating cytokines. Activated macrophages change their cell morphology and secretion pattern, and are characterized by phagocytosis. The phagocytosis is an essential step in various macrophage functions, including tissue remodeling, the decomposition of foreign particles, and the continuous removal of unwanted or dying cells (Shapouri-Moghaddam et al. 2018; Ma et al. 2019). In this study, RAW 264.7 cells cultured in normal medium were rounded with normal morphology. However, after treatment with I6CA, the cells turned into a typical active macrophage morphology that could aid in phagocytic uptake by increasing the contact area with outer substances. More importantly, the phagocytic capacity was significantly increased by I6CA treatment. The phagocytic capacity of the cells treated with 0.5 ng/ml LPS was similar to that of cells treated with 100 μM I6CA. These results indicate that I6CA in RAW 264.7 cells can at least act as a macrophage activator that significantly improves the phagocytic uptake ability. LPS used as a positive control is known to be an effective stimulator of the immune system and has been widely applied to models for inducing macrophage activation (Jiménez-Dalmaroni et al. 2016; McCall et al. 2020). Therefore, our results predict that the mode of action of I6CA may be similar to that of LPS.

Along with phagocytosis, activated macrophages release cytotoxic and immune mediators. Among them, the overproduction of NO is mainly on account of high expression of iNOS in the NOS isoform, and COX-2 is involved in the synthesis of PGE_2_ from arachidonic acid. NO and PGE_2_ are important mediators of inflammation, as well as the immune response, and are responsible for the regulation of host innate immune responses. Since they can also improve the phagocytosis and lysis of macrophages (DeNardo and Ruffell 2019), the increased synthesis of NO and PGE_2_ is an indicator of macrophage activation. The present results indicated that I6CA significantly stimulated the production of NO and PGE_2_, which was associated with increased expressions of iNOS and COX-2 at the transcriptional and translational levels. As expected, all results were similar in the LPS-treated RAW 264.7 cells, which indicated that the increased production of NO and PGE_2_ in the I6CA-treated cells was due to the enhanced expression of iNOS and COX-2.

Macrophages produce various cytokines to regulate the balance of inflammatory and immune responses. Among them, early pro-inflammatory cytokines, including TNF-*α*, IL-1*β* and IL-6, produced by activated macrophages, have multiple functions in the inflammatory process and also promote the production of other cytokines. They also enhance the immune function of macrophages because they are multifunctional cytokines that play a key role in rejecting tumor cells and activating T cells. Moreover, the secretion of anti-inflammatory cytokines, including IL-10, IL-12 and transforming growth factor-*β*, is necessary for the immune response, as sustained release of pro-inflammatory cytokines can negatively affect the repair of damaged tissue (Batlle and Massagué 2019; Domínguez-Andrés et al. 2020). In this work, the secretion of cytokines, such as TNF-*α*, IL-1*β*, IL-6 and IL-10, was significantly increased in I6CA-treated RAW 264.7 cells. Consistent with this, their mRNA and protein levels also increased, and the LPS-treated group showed a similar tendency. Therefore, our findings indicate that the ability of I6CA to enhance macrophage function may be associated with the increased production of immune cytokines.

TLRs are known to act as an integral role in the innate immune response as well as the induction of inflammatory responses by host cell recognition and specific patterns of microbial pathogens (Jiménez-Dalmaroni et al. 2016; McCall et al. 2020). Among TLRs, TLR4 plays a key role in the inflammatory and immune responses and activates TNF receptor-associated factor 6 (TRAF6) through the MyD88-dependent pathway, one of the adapter molecules. TRAF6 further serves as an important mediator of signal transduction pathways that regulate immunomodulatory-related downstream gene expression by promoting the activation of NF-κB and MAPKs signaling pathways (Walsh et al. 2015; Tang et al. 2018). Therefore, we speculated that I6CA could activate the TLR4 signaling pathway, and observed that I6CA significantly upregulated the expression of Myd88 as well as TLR4 in RAW 264.7 cells, which was similar to that of the LPS-treated cells. I6CA also increased the complex formation of TLR4 and MyD88, indicating I6CA could enhance the association of TLR4 with its adaptors, leading to activation of TLR4. Lys89, Arg90, Lys91, Lys122, Lys125, Lys128 and Lys132, which are reported to essential region for LPS binding to TLR4/MD2 complex and for NF-κB activation (Kim et al. 2007). In present study, a molecular docking study was carried out to investigate the interaction with I6CA and TLR4/MD2 complex. Our finding showed that I6CA have highly binding affinity with TLR4/MD2 complex, and I6CA was structurally predicated to interact with Lys 132 residue of TLR4/MD2 complex where is an LPS binding site. Based on these results, we considered that I6CA has sufficient potential to activate the TLR4 signaling pathway.

The NF-κB signaling pathway functions as an essential messenger of macrophage activation that regulates many immune regulatory mediators (Beinke and Ley 2004; Dorrington and Fraser 2019). Inactive NF-κB dimer (p65/50) interacts with IκB-*α* in the cytosol to form complexes. As IκB*α* is phosphorylated and degraded subsequently through the ubiquitin-proteasome system, the NF-κB dimers are released from the NF-κB/IκB-*α*-complex and translocate to the nucleus to initiate transcriptional activity of the target genes. This is a typical NF-κB activation pathway that occurs through the formation of signaling complexes including MyD88 and TRAF6 when LPS is recognized by the TLR4 receptor in the plasma membrane (Tang et al. 2018; Li et al. 2019). According to our results, the expression of NF-κB and IκB-*α* was greatly decreased in the cytoplasm of RAW 264.7 cells upon I6CA stimulation compared to the control group. However, the expression of NF-κB in the nucleus was markedly increased by treatment with I6CA. Similar trends were also found in LPS-treated RAW 264.7 cells. Therefore, the data indicated that the liberated NF-κB was translocated from the cytoplasm into the nucleus as IκB-*α* degraded in I6CA-treated cells, similar to the mode of action of LPS, demonstrating that I6CA has an immunomodulatory effect by increasing the production of immunomodulatory mediators and cytokines through activation of the TLR4-mediaetd NF-κB signaling pathway in RAW 264.7 cells. However, additional studies are needed to determine the link between TLR4 and the NF-κB signaling pathway and other signaling pathways that may be involved in the immune effects of I6CA. Nevertheless, the present results could provide the first information needed to understand the mechanism of the immunomodulatory potential associated with macrophage activation by I6CA.

In summary, our data indicate that the phagocytic capacity of RAW 264.7 cells was clearly improved after treatment with I6CA, and the cell morphology changed correspondingly. The production of immunomodulatory mediators and cytokines was increased, which might be due to the increased expression of their respective genes by I6CA. Moreover, the underlying mechanism of the I6CA-mediated activation of macrophages was related to the activation of the TLR4-mediaetd NF-κB signaling pathway. Therefore, the I6CA used in this study could potentially be used as a candidate to increase immune activity.
